# Simultaneous Assessment of Chicken Freshness and Authenticity Using a Single Multispectral Imaging Device: A Cross-Laboratory Evaluation Using Identical Instruments

**DOI:** 10.3390/s26092702

**Published:** 2026-04-27

**Authors:** Anastasia Lytou, Maria-Konstantina Spyratou, Aske Schultz Carstensen, George-John Nychas, Nikos Chorianopoulos

**Affiliations:** 1 Laboratory of Microbiology and Biotechnology of Foods, Department of Food Science and Human Nutrition, School of Food and Nutritional Sciences, Agricultural University of Athens, Iera Odos 75, 11855 Athens, Greece; alytou@gmail.com (A.L.); maritinaspy@gmail.com (M.-K.S.); gjn@aua.gr (G.-J.N.); 2 Videometer A/S, Hørkær 12B 3, DK-2730 Herlev, Denmark; asc@videometer.com

**Keywords:** meat quality aspects, spoilage, microorganisms, portable sensor, spectral imaging, identical instruments

## Abstract

**Highlights:**

**What are the main findings?**

- Multiple quality parameters can be evaluated in a single analysis.
- A portable MSI device can assess chicken freshness and authenticity.

**What are the implications of the main findings?**

- Diverse data sources are essential for improving prediction accuracy.
- Informative wavelengths can be leveraged across analytical technologies.

**Abstract:**

This study evaluated a portable multispectral imaging (MSI) system for simultaneously assessing chicken meat quality, including freshness and authenticity detection. For freshness, total aerobic counts and MSI analyses were performed on fresh and thawed samples throughout storage at 4 °C. For authenticity (product condition and origin), Greek and Danish chicken samples, both fresh and thawed, were analyzed in separate laboratories using identical instruments. Data were modeled using PLS-R, kNN, and SVM. Model performance for total viable count prediction was evaluated via R^2^ and RMSE, while classification used accuracy, specificity, recall and precision. PLS-R beta coefficients highlighted the contribution of specific wavelengths. For Greek chicken fillets, kNN achieved the best performance on fresh samples (RMSE = 0.347, R^2^ = 0.979), while PLS-R performed best on thawed samples (RMSE = 0.787, R^2^ = 0.859). Wavelength 460 nm was the most important for all freshness predictions. Differences between Danish and Greek samples were observed in classification performance, optimal algorithms and key wavelengths. For origin classification (using fresh and thawed samples), models reached near-perfect accuracy, with PLS-DA highlighting 660 nm and 850 nm as most significant. These results demonstrate the MSI system’s potential for the rapid, accurate and simultaneous evaluation of multiple chicken meat quality attributes using a single instrument.

## 1. Introduction

The continuous consumer interest in meat, along with recent dietary trends favoring protein-rich foods, makes it a particularly popular food category for which maintaining high-quality standards is of great importance. Global poultry consumption is projected to account for approximately half of the increase in total meat consumption, with poultry expected to provide around 43% of the protein derived from all meat sources by 2033 [1].

For these reasons, it is essential to improve the methods used to assess product quality through the development of advanced quality management systems that integrate conventional analytical techniques, sensors and analytical platforms. In this context, the development of non-invasive, rapid and accurate detection techniques based on portable instruments, which can be applied throughout the food chain, is of critical importance in meeting the requirements of such systems [2,3]. These technologies may produce large amounts of data that can be processed using machine learning approaches to make the determination of the use-by date a more product-specific and dynamic procedure, as well as to assess the origin of a food product or detect fraudulent practices [4,5].

Among the technologies extensively investigated in recent years, spectral imaging techniques, including hyperspectral and multispectral imaging, have been widely used to address issues related to meat freshness, fraud and authenticity. Between the two, multispectral imaging (MSI) is particularly suitable for samples that contain both spectral and spatial information, such as biological materials with complex quality attributes. Although hyperspectral imaging provides more detailed spectral information than MSI, its acquisition time, complexity and cost are generally higher [6]. Therefore, MSI, which relies on selected characteristic wavelengths, represents a promising alternative for specific applications in the meat industry [7].

Another critical factor for the successful application of these methods is the use of portable instruments of relatively simple design that offer performance comparable to benchtop systems while providing greater flexibility in use. In addition to being handheld and usable in various settings, such devices are typically available at a lower cost [8]. Further to appropriate instrumentation, the complex and multidimensional nature of the data generated by these analyses necessitates the application of suitable statistical or machine learning methods to fully exploit the data and derive reliable results that support sound decision-making. Selecting the most effective pattern recognition or machine learning technique for a given analytical platform is often challenging and requires a comparative evaluation of different algorithms to achieve optimal predictive performance [9].

In the current study, as is often reported in the literature, the algorithms used include Partial Least Squares Regression/Partial Least Squares Discriminant Analysis (PLS-R/PLS-DA), Support Vector Machine (SVM) and k-Nearest Neighbors (kNN) for both regression and classification tasks. Partial Least Squares (PLS) methods are widely applied due to their ease of use, even by non-specialists. However, algorithms such as SVM and kNN are also implemented in user-friendly statistical software and offer alternative data processing approaches. SVM operates by mapping input data into a high-dimensional space to construct an optimal separating hyperplane, with the final model defined by a limited number of support vectors, enhancing robustness and efficiency. In contrast, kNN classifies samples based on the majority class among their nearest neighbors and is particularly suitable for pattern recognition tasks [10,11]. Overall, the application of machine learning tools holds significant potential for the food industry by improving efficiency, reducing food waste and ensuring product quality and authenticity.

Finally, even with the selection of appropriate instrumentation and the optimal algorithm, the success of a method ultimately depends on its ability to perform consistently when transferred across different working environments, whether in industrial or laboratory settings. This highlights the necessity for analytical techniques to be applicable across diverse laboratory conditions without compromising repeatability and accuracy—an ongoing challenge in analytical chemistry, particularly in food analysis [12]. For this reason, when evaluating the robustness of a method, it is important to account for variability arising from different analytical environments, instruments and/or analysts during both sample collection and analysis [11].

The primary objective of this study was to explore the capabilities of a portable MSI device for determining freshness, as well as authenticity related to the physical condition of the chicken samples (fresh or thawed state) and their origin, which may constitute a potential case of food fraud. The study also aimed to identify the wavelengths that could effectively address all three aspects mentioned above, as indicated by the standardized coefficients derived from the respective analyses. The main contribution of this study, compared with previous studies assessing MSI applications, lies in the evaluation of this MSI analysis using samples from multiple batches/lots of chicken originating from different geographical regions and analyzed using different devices based on the same technology. In addition, the analyses were conducted in different laboratories to account for potential variability arising from distinct experimental conditions or materials. Furthermore, three commonly used algorithms were evaluated for their effectiveness in addressing each of the aforementioned scenarios.

## 2. Materials and Methods

### 2.1. Experimental Design

**Figure 1:**
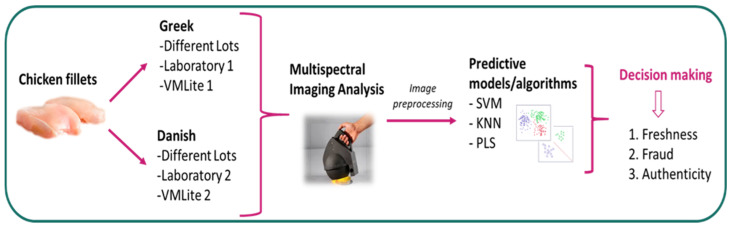
A schematic representation of the experimental design. VMLite: the portable MSI instrument used for the analyses.

The experimental design is illustrated in Figure 1.

The process was divided into two parts. The first part was carried out at the Laboratory of Food Microbiology and Biotechnology of the Agricultural University of Athens (AUA), while the second part was conducted in Herlev (Copenhagen, Denmark), at the laboratories of Videometer, by personnel from the AUA.

During the first stage, aerobically packaged chicken breast fillets of Greek origin and different lots were purchased (*n* = 180). The samples originated from three batches (Batch 1, 2, and 3). The first two batches corresponded to samples from the same company but with different production dates, while the third batch consisted of samples from a different company (Table 1). The samples were analyzed both microbiologically (determination of total aerobic bacteria) and using the multispectral imaging method, as fresh (as purchased) and during storage at 4 °C for a period of 6 days. Analyses were performed—for each batch separately—at 0, 1, 2, 4, 5, and 6 days of storage (six time points in total).

A subset of the initial samples (*n* = 84) was frozen at −18 °C for 48 h and subsequently thawed at 4 °C for 6–8 h. These samples were all thawed simultaneously (per batch) and then processed and analyzed following the same procedure as fresh samples (i.e., analyses conducted at the same six storage time points), with a two-day offset due to the prior freezing and thawing process. For these samples, day 0 was defined as the day immediately after thawing.

In the second stage, a similar procedure to the first part was followed with the difference that Danish-origin chicken fillets were used (*n* = 150 (75 fresh and 75 frozen/thawed)) (Table 1). During this phase, only multispectral imaging analysis was carried out as the Videometer laboratory lacked appropriate facilities for microbiological analyses. Another difference between the two experimental parts was the utilization of different multispectral imaging devices (different items) albeit with the same technology and capabilities.

During the data analysis stage, several scenarios were examined, including the prediction of freshness levels (i.e., estimation of microbial populations), the discrimination between fresh and thawed samples using either the combined dataset (Greek and Danish) or individual datasets, as well as the determination of sample origin using fresh, thawed, or combined datasets.

### 2.2. Microbiological Analysis

For microbiological analysis, 15 g of fresh chicken breast was aseptically transferred into a sterile homogenization bag (BagLight^®^, INTERSCIENCE, Paris, France). Subsequently, 135 mL of Ringer’s solution (LAB M Limited, Lancashire, UK) was added to obtain a 1:10 dilution. The samples were homogenized using a Stomacher (Lab Blender 400, Seward Medical, London, UK) for 60 s at room temperature. Serial decimal dilutions were then prepared, and 0.1 mL aliquots were spread onto Plate Count Agar (PCA; Tryptic Glucose Yeast Agar, Biolife, Milan, Italy) using the surface plating technique. Plates were incubated at 30 °C for 48 h, after which colonies were counted and results expressed as log CFU/g. Total aerobic counts (TACs) were determined daily over a period of 6 days. Mean values and standard deviations were calculated.

### 2.3. Multispectral Imaging Analysis

Multispectral imaging data were acquired using portable Videometer devices (VIDEOMETER, Herlev, Denmark). Measurements in the two laboratories were performed using different units of the same system, with identical sensor technology, acquisition principles, and spectral configuration. The Videometer system is based on sequential illumination of the sample at discrete wavelengths inside an integrating sphere, which provides uniform diffuse lighting and standardized image acquisition conditions. The resulting monochromatic images are combined into a multispectral dataset in which each pixel contains wavelength-dependent reflectance information [13]. Before imaging, each instrument was calibrated using the standard white reference plate. Image acquisition and processing were performed using VideometerLab software (v. 3.22).

The instrument utilized in this study is a portable, wireless device designed to capture images across 7 wavelengths from 405 nm to 850 nm (405 nm (violet), 460 nm (blue), 525 nm (cyan), 590 nm (amber), 621 nm (red), 660 nm (red), and 850 nm (near-infrared—NIR)). This device captures images in 7–10 s, enabling quick in-field analysis. The instrument was further designed and developed during the TMF project (ERA-NET Cofund BlueBio, Grant Agreement No. 817992) [4], while additional applications of this version were explored in the present work.

### 2.4. Data Analysis

To assess freshness and evaluate the two scenarios related to the authenticity of the chicken samples, the collected spectral data were further analyzed.

At first, the relative standard deviations (RSDs) of the reflectance values of the initial samples used in the analyses were calculated in order to evaluate the extent of the initial variability in the dataset.

For model development and validation, the dataset was divided into two subsets: a training set and a test set, using a 70:30 ratio. A part of the training data (15%) was used for internal validation of the developed model. Analyses were performed using individual datasets for each case. Three algorithms—PLS, SVM, and kNN—were evaluated using either regression or classification approaches, depending on the objective. For TAC prediction, model performance was assessed using the coefficient of determination (R^2^) and Root Mean Square Error (RMSE). In multispectral imaging, R^2^ is commonly used to evaluate model fit, while RMSE reflects the deviation between predicted and measured values; thus, a robust model is characterized by high R^2^ and low RMSE values. However, performance thresholds may vary depending on the technique and application [6]. For classification tasks, model performance was evaluated using accuracy, specificity, recall and precision.

The models were created using the XLStat 2021.1 software (Addinsoft, Paris, France) analysis and statistics software embedded in Microsoft Excel, while the data did not receive any further pre-processing.

## 3. Results and Discussion

### 3.1. Microbiological Quality

**Figure 2:**
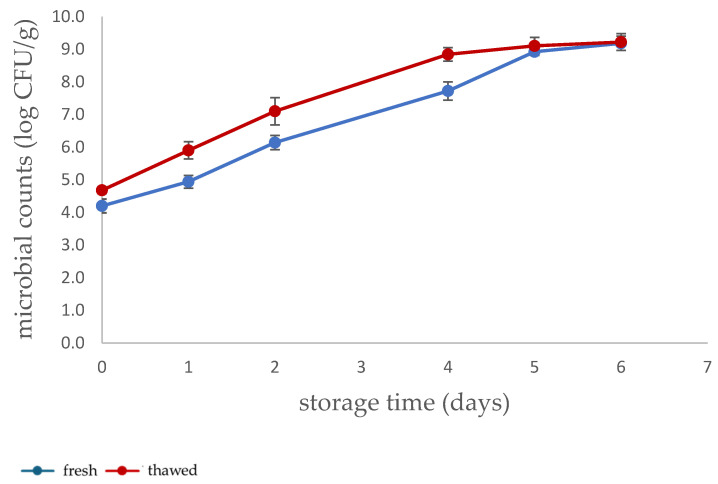
Total aerobic counts (TACs) in fresh and thawed chicken fillet samples.

The microbial population of the TACs on the surface of chicken fillets for each sample condition (fresh or thawed) is presented in Figure 2. The initial load of the TACs was 4.2 and 4.7 log CFU/g in fresh and thawed samples, respectively. The initial population of total aerobic microorganisms is slightly increased in thawed samples, although they were processed according to the refrigerator thawing protocol reported [14]. The thawing period (5–6 h at 4 °C) required for the samples likely contributed to this slight increase in the initial microbial populations. Similarly, a more pronounced growth of microorganisms in thawed samples has been observed throughout storage. Freezing and thawing cause tissue damage and fluid loss, generating nutrient-rich microenvironments that facilitate microbial growth [15]. By the end of the storage period, the total aerobic counts were the same (9.2 log CFU/g) in both groups.

### 3.2. Estimation of Microbiological Quality (Freshness) Using MSI Data

As also mentioned in Section 2, the present study included samples with significant variability, starting from the initial samples, where fresh chicken fillets of different geographical origins were used in the analyses. The three different lots corresponded to samples of the same brand but with different production dates (Batches 1 and 2), while Batch 3 consisted of products from a different company.

Table 2 presents the levels of variability—expressed as RSD (%)—for the initial samples (prior to freezing and storage, and thus before spoilage). Across the different wavelengths, the RSD values ranged approximately from 2 to 11% for the Danish samples and from 2 to 8% for the Greek samples, while the mean RSD across all wavelengths was 6.15% and 4.97% for the Danish and Greek samples, respectively. Notably, a nearly gradual decrease in RSD is observed in the Danish samples with the increasing wavelength (from 405 to 850 nm), a trend that is not evident in the Greek samples.

**Figure 3:**
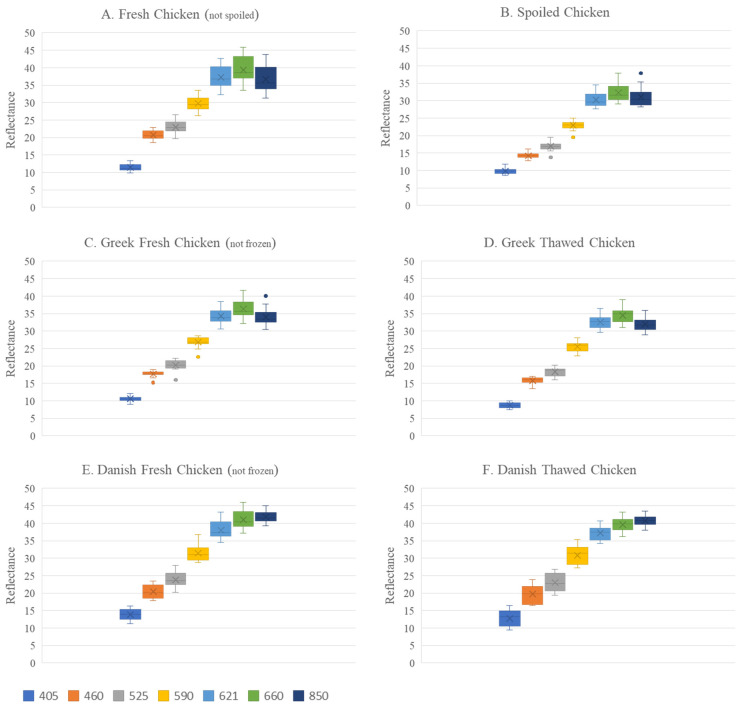
Range of reflectance per wavelength in Greek fresh (not spoiled) (**A**) and spoiled (**B**) chicken samples, in Greek fresh (not frozen) (**C**) and thawed (**D**) samples as well as in Danish fresh (not frozen) (**E**) and thawed (**F**) samples.

The higher heterogeneity observed in the Danish samples is also reflected in Figure 3, where variability arising not only from the initial samples but also from freezing/thawing and spoilage progression is included. In both Figure 3E,F (Danish samples), more pronounced variation in reflectance across wavelengths is clearly observed compared to the Greek samples (Figure 3C,D). Furthermore, based on Figure 3A,B, a clear difference is observed in the spectra of spoiled samples (TAC > 7.0 log CFU/g) compared to fresh samples (TAC < 5.5 log CFU/g), with spoiled samples exhibiting lower variability and reduced reflectance values.

**Figure 4:**
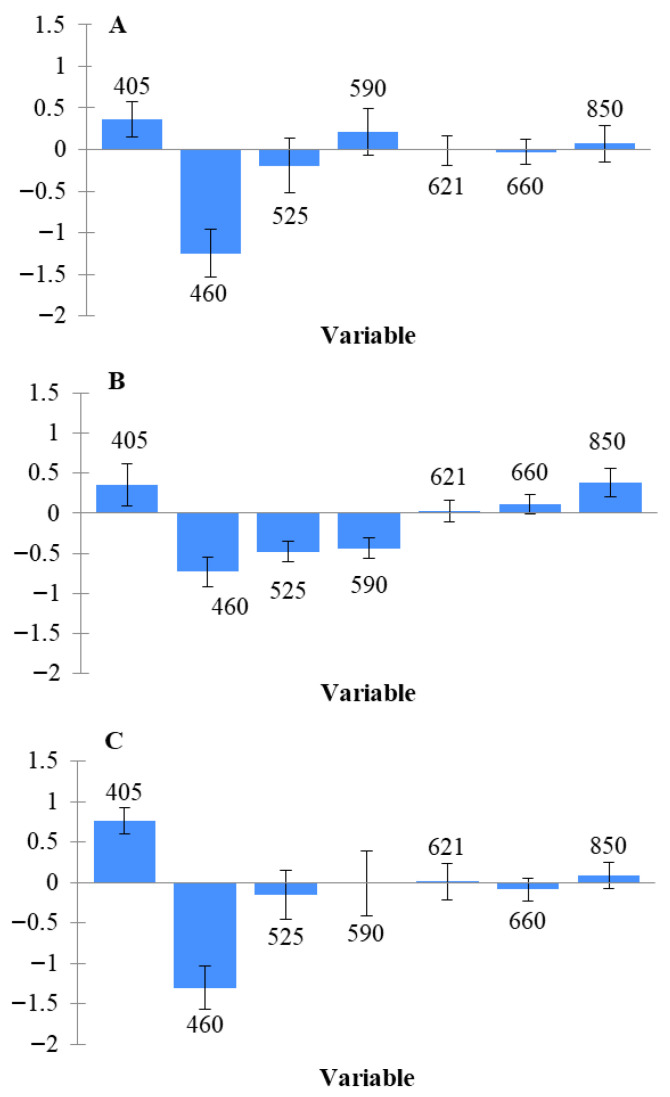
Significant bands (standardized coefficients—PLS-R) for the prediction of microbial counts in chicken fillets: fresh (**A**), thawed (**B**), combination of them (**C**).

Model performance for estimating the TACs using MSI data analysis in Greek chicken fillets (fresh, thawed, and their combination) is presented in Table 3. For the fresh (non-frozen/thawed) samples, the RMSE values for prediction ranged from 0.347 to 1.015, while the R^2^ values ranged from 0.816 to 0.979. The best performance was achieved using the kNN algorithm, with an RMSE of 0.347 and an R^2^ of 0.979. It is worth noting that PLS-R and SVM also demonstrated very good performance. In contrast, for the prediction of microbial counts in thawed samples, the best performance was achieved by the PLS-R algorithm, with RMSE and R^2^ values of 0.787 and 0.859, respectively. Additionally, for the combined dataset of fresh and thawed samples, PLS-R again achieved the highest performance, with RMSE and R^2^ values of 0.834 and 0.863, respectively. Overall, PLS-R appears to be an effective algorithm for predicting microbial counts using spectral data, as also reported in previous studies [16,17]. In addition, although the kNN algorithm showed very strong performance in the present study, it has not been widely applied in the literature for this specific purpose. Moreover, the beta coefficients of the PLS-R model are presented to illustrate the contribution of individual wavelengths to model development (Figure 4). Among the wavelengths used in the analysis, 460 nm was identified as the most informative variable across all three datasets (fresh, thawed, and combined), based on the beta coefficient values. Additionally, 405 nm also contributed to the performance of the PLS-R model. A greater diversity of significant wavelengths was observed in the thawed samples, where 850 nm also appeared to play a role in estimating microbial populations. The greater variation in frozen–thawed samples is likely due to mechanical tissue damage from freezing and uneven water loss during thawing [18]. The significance of specific wavelengths, particularly 405 and 460 nm, has also been reported in previous studies [5,19]. Given that the meat was stored under aerobic conditions, Pseudomonas spp. is expected to be the dominant microbial group associated with product spoilage, while other microorganisms such as Enterobacteriaceae and lactic acid bacteria also contribute significantly. Considering that common spoilage microorganisms in meat, such as Pseudomonas and Enterobacteriaceae, produce fluorescent compounds (e.g., porphyrins and siderophores) [20,21], the importance of wavelengths in the near-ultraviolet region can be explained.

Furthermore, the blue region (460 nm) has been associated with Nicotinamide Adenine Dinucleotide (NADH) and flavins (intrinsic chromophores), which also reflect metabolic activity and subsequently spoilage processes [22,23]. Finally, the importance of 850 nm (located on the slope of water absorption) in the spoilage of frozen–thawed samples is likely related to modifications in meat tissue structure during freezing and thawing, as well as the gradual water loss that occurs during storage, since thawed meat exhibits reduced water-holding capacity [24]. These physicochemical changes are closely associated with meat quality deterioration and create conditions that facilitate microbial growth during storage.

In another approach for assessing freshness, samples were classified based on microbial population levels, using 6.0 log CFU/g as the threshold. According to previous studies, a population of 6.0 log CFU/g is considered a level at which chicken products are not yet spoiled (when stored under aerobic conditions) or are in an early stage of spoilage [25,26].

**Figure 5:**
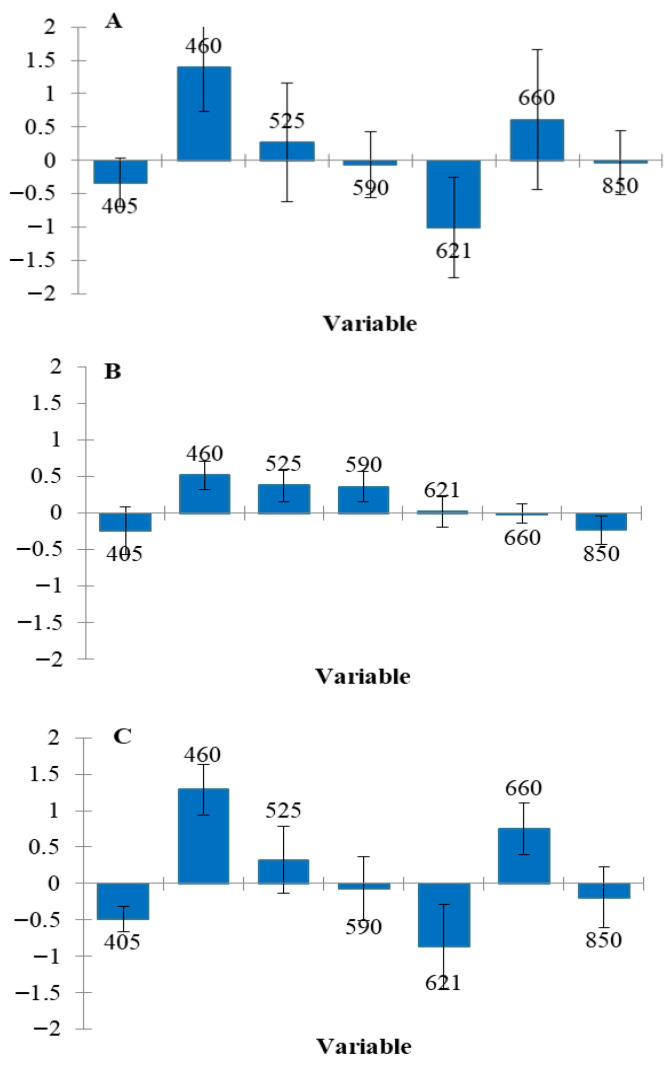
Significant bands (standardized coefficients—PLS algorithm) for the prediction of freshness in chicken fillets: fresh (**A**), thawed (**B**), combination of them (**C**).

Table 4 shows the performance metrics for classifying the samples into two classes (fresh/spoiled). For fresh (not frozen) samples, all algorithms achieved high accuracy, ranging from 96.43% to 96.45%, while the values for precision, recall and specificity were also high. For thawed samples, accuracy ranged from 90% to 95%, while for the combined dataset, the SVM algorithm achieved the highest performance with 100% accuracy. Although this classification analysis has limitations in precisely determining microbial spoilage, it has important implications in cases where distinguishing between fresh and non-fresh or spoiled samples is challenging. Moreover, combining the two approaches can serve as confirmation, significantly enhancing the reliability of the analysis. Consequently, less fresh samples may reach the market more quickly, or, if already distributed, be assigned shorter expiration dates. The most informative variable for all predictions (fresh, thawed, and combined) was 460 nm, as its beta coefficient was notably higher than those of other wavelengths (Figure 5). Other wavelengths influencing the PLS-DA model included 405 nm, 621 nm, and 660 nm for fresh and combined samples, and 525 nm and 590 nm for thawed samples. As shown, both in TAC prediction through regression and in freshness classification, 460 nm consistently emerged as the most significant. However, due to the nature of this supervised, two-class classification analysis, additional wavelengths also contributed valuable information.

### 3.3. Discrimination of Fresh and Frozen/Thawed Chicken Fillets—Mislabeling of Product Condition (Fresh vs. Thawed)

A unique characteristic of this analysis is the acquisition of data from different laboratories, devices and countries. Additionally, the samples were highly diverse in each case, reflecting variations in quality status (fresh, semi-fresh, spoiled) and sample condition (fresh or thawed).

The performance of the selected models for classifying the samples (Greek, Danish, or combined) into quality classes (fresh or thawed) using MSI data is presented in the confusion matrices for PLS-DA, SVM, and kNN (Table 5). For Greek chicken fillets, the PLS-DA model achieved the highest accuracy of 76.09%. Overall, model performance for the Danish fillets was higher, with the SVM algorithm reaching the highest accuracy of 95.00%. In addition, samples of Greek origin showed low precision and specificity across all the applied algorithms.

One possible reason for the disparity in algorithm performance between Greek and Danish fillets is the defrosting process, which was conducted using different equipment in the two laboratories. Factors such as airflow and the precise positioning of samples in the refrigerator during thawing could influence the extent of physicochemical changes in the meat tissue. Moreover, Danish fillets may have contained more fat or higher myoglobin content (darker red color) due to differences in rearing practices and diet. These factors can lead to distinct differences between fresh and thawed chicken, as they are significantly affected by the freeze–thaw cycle [27]. For the combined dataset, the SVM algorithm achieved the highest accuracy of 75.76%. Both the PLS-DA and SVM models appeared to be more effective, as reported in previous studies on similar topics [5,28]. In this case, the variability arising from sample spoilage may have significantly affected the model’s performance, as even in fresh samples, the characteristics of a non-spoiled thawed chicken (e.g., differences in moisture) may overlap with certain features associated with spoilage.

**Figure 6:**
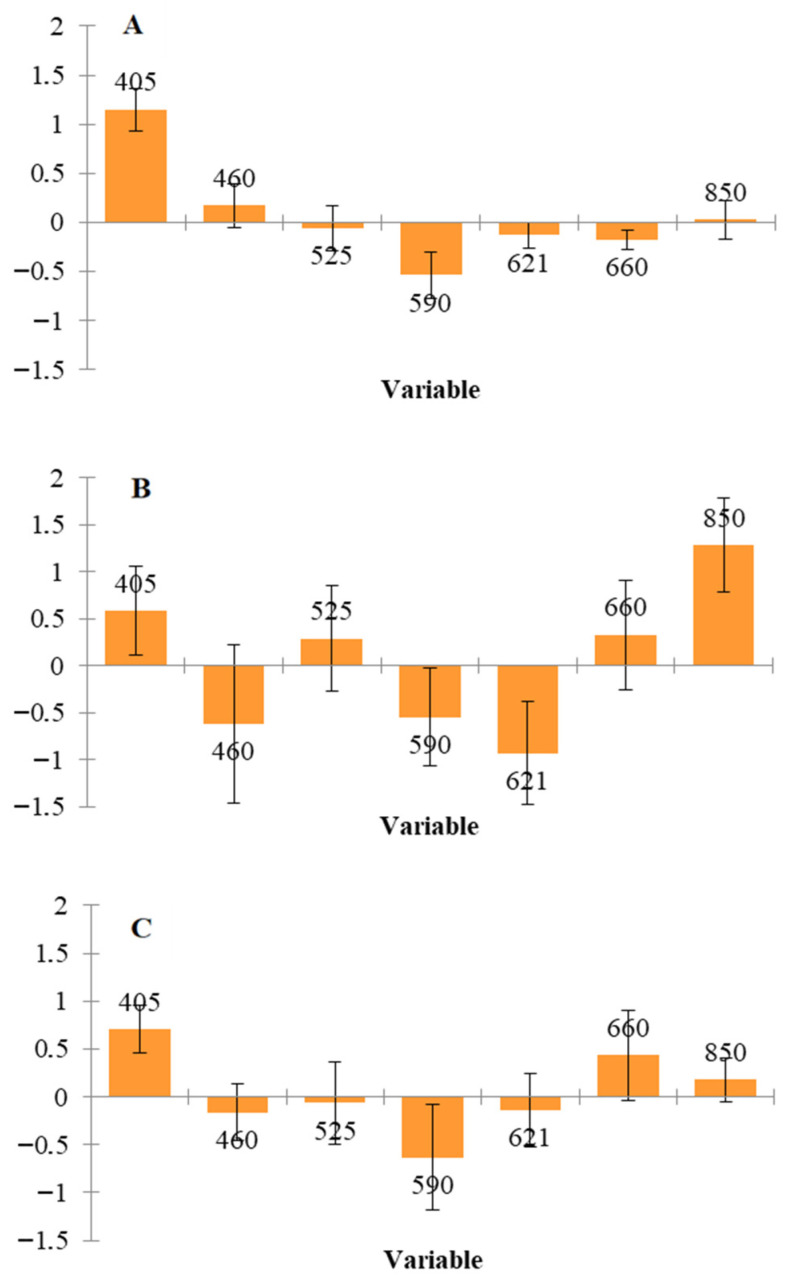
Significant bands (PLS) for the discrimination of fresh and thawed chicken fillets of Greek (**A**) and Danish (**B**) origin as well as combination of them (**C**).

During freezing and thawing, ice crystal formation disrupts muscle structure, promoting the release of non-heme iron and increasing oxygen availability. This accelerates the oxidation of deoxymyoglobin and oxymyoglobin to metmyoglobin, while simultaneously catalyzing lipid oxidation, creating a synergistic cycle that affects meat color and overall quality. These mechanisms are directly reflected in the observed spectral changes: variations in the 505–630 nm region are associated with alterations in meat color due to myoglobin oxidation, whereas bands in the near-infrared region (e.g., 850–940 nm) relate to moisture loss and enhanced lipid oxidation [27,29,30,31]. This explains why, based on the beta coefficients (Figure 6), wavelengths at 405 nm and 590 nm were the most informative for distinguishing fresh from frozen/thawed samples, with additional discriminative contributions at 621 nm and 850 nm for the Danish fillets.

### 3.4. Discrimination of Chicken Fillets by Origin: Greek vs. Danish—Mislabeling Related to Origin

As in the previous analysis, the diversity in this case is also high, since samples from all levels of spoilage, as well as fresh and frozen/thawed samples, were used for discrimination based on origin. Different countries have unique agricultural practices and cultural habits that significantly affect animal growth and metabolic profiles. Notable differences in metabolic patterns may arise from intensive farming or from organic farming, where animals are raised with minimal chemical interventions, leading to slower growth and more natural metabolic profiles. Consequently, these differences can be reflected in spectral data, producing characteristic profiles that allow discrimination of products based on their origin.

**Figure 7:**
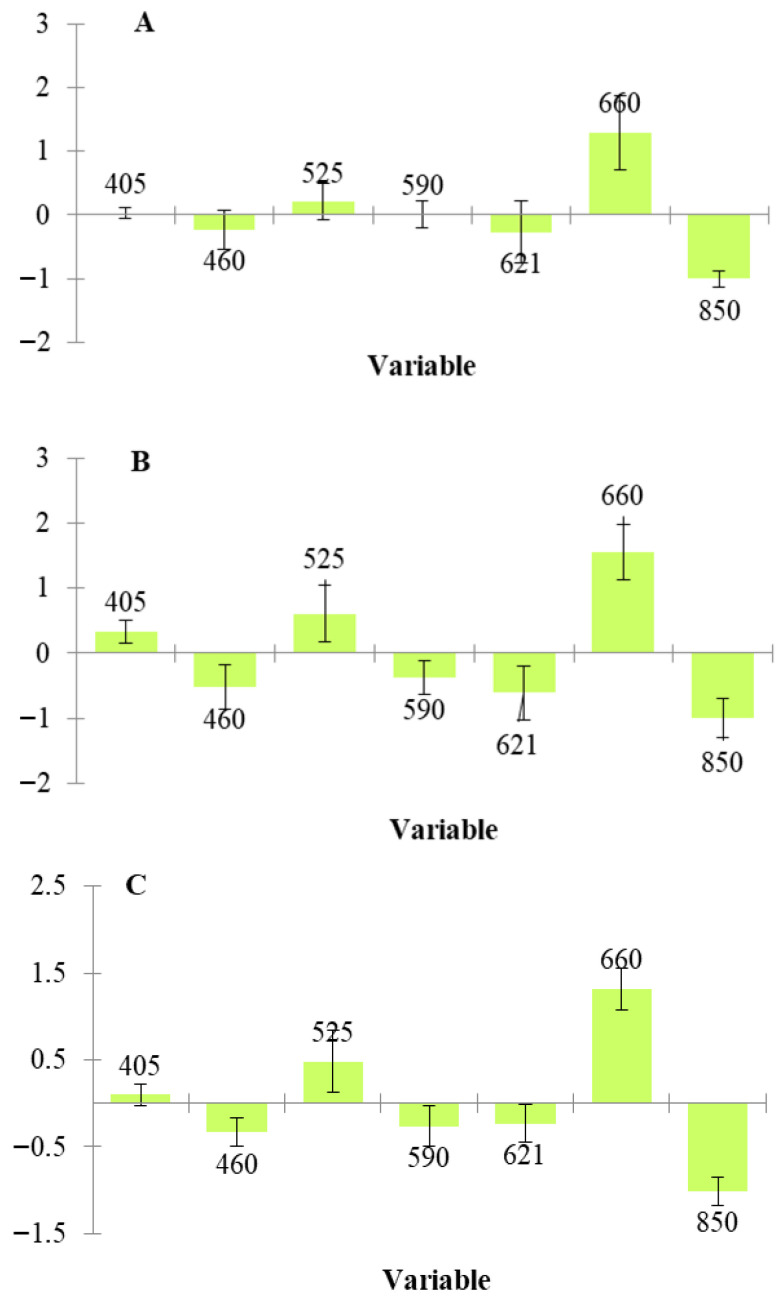
Significant bands (PLS algorithm) for the prediction of origin in chicken fillets: fresh (**A**), thawed (**B**), combination of them (**C**).

Table 6 presents the performance metrics for classifying the samples (fresh, thawed, and combined) according to their origin (Greek or Danish). The models demonstrated very high performance, with all accuracies, except one, reaching 100.00%. Additionally, Figure 7 shows that, across all PLS-DA analyses, the most significant wavelengths were 660 nm and 850 nm. This indirectly supports the previous analysis, suggesting that Danish chicken may have a higher pigment content (myoglobin) or greater fat levels, characteristics that also contributed to their differentiation from Greek chicken.

Overall, this study further confirms the findings of previous research [27,32,33] regarding the ability of spectral analysis to evaluate multiple quality-related parameters, including freshness, origin determination, and the detection of potential fraud involving the sale of frozen/thawed chicken instead of fresh products. Furthermore, the consistency of the models’ success despite such diversity is remarkable, especially considering the challenges reported in the literature regarding reduced performance when analyses are conducted using different instruments or in different settings (laboratory or industry) [27,32].

### 3.5. Limitations and Further Steps for the Application of This Approach in Real Conditions

Although this study demonstrates satisfactory results regarding the use of MSI for the simultaneous assessment of multiple meat quality parameters at relatively low cost, there are several aspects that require further investigation for its application under real-world conditions. The first aspect concerns the validation of its effectiveness throughout the cold chain. Particularly for freshness assessment, it is well known that temperature fluctuations during transportation and distribution affect both the characteristics and the rate of spoilage. Moreover, despite its successful testing across different environments/laboratories, further validation under field conditions (e.g., in trucks, storage facilities, retail environments, etc.) would provide additional confirmation of its applicability.

A second aspect relates to evaluating this technology across a wider range of chicken products in order to achieve broader use. This could include different product types as well as various packaging conditions, which may influence the underlying mechanisms of spoilage and quality deterioration. The third aspect, particularly concerning the assessment of authenticity, involves increasing dataset variability. For the evaluation of physical condition (fresh vs. thawed), it would be meaningful to further investigate different freezing and thawing conditions, especially in terms of time, temperature, and sample size. With regard to authenticity of origin, it is important to include additional origins or, if focusing on a specific origin, to incorporate more brands and batches (lots) from the same origin to enhance the reliability of the results. Finally, to further optimize the predictions, a wider range of algorithms (e.g., Random Forest, XGBoost, Artificial Neural Networks) could also be explored, as they may prove useful for similar tasks.

## 4. Conclusions

This study represents a further step toward the utilization of portable spectral imaging instruments for the assessment of different parameters related to meat quality, demonstrating satisfactory results in most of the cases investigated. It is noteworthy that, for each individual analysis, samples with high variability were used, including differences in freshness level, origin, or sample condition (fresh or thawed), depending on the analysis. This variability was further enhanced by the fact that measurements for the two quality parameters were performed in different laboratories using different instruments (though of the same type). Finally, the identification of specific wavelengths that contribute to the discrimination of samples, depending on the respective objective, is considered particularly important, as they can be further utilized by researchers for the development of even more efficient instruments.

## Figures and Tables

**Table 1:** Lots and number of samples used in the analysis.

Origin/Lots	Condition	No of Samples
Greek		
Batch 1	Fresh	56
	Frozen/Thawed	40
Batch 2	Fresh	23
	Frozen/Thawed	24
Batch 3	Fresh	17
	Frozen/Thawed	20
Danish		
Batch 1	Fresh	38
	Frozen/Thawed	30
Batch 2	Fresh	18
	Frozen/Thawed	23
Batch 3	Fresh	19
	Frozen/Thawed	22

**Table 2:** Reflectance, standard deviations (stdev) as well as relative standard deviations (RSD%) across different batches of Danish and Greek fresh fillets.

Danish							
Wavelengths	405	460	525	590	621	660	850
Batch 1							
Reflectance	15.56	22.79	26.53	35.49	42.21	44.74	44.04
stdev	1.13	1.14	1.46	2.01	1.94	1.91	1.22
RSD (%)	7.26	5.02	5.49	5.68	4.59	4.27	2.76
Batch 2							
Reflectance	15.19	22.69	26.56	35.76	42.60	44.91	44.13
stdev	1.33	1.46	1.87	2.45	2.43	2.51	1.61
RSD (%)	8.76	6.42	7.05	6.84	5.70	5.58	3.65
Batch 3							
Reflectance	13.02	21.10	24.75	33.52	41.55	43.87	43.89
stdev	1.45	2.02	2.77	2.82	1.70	1.49	1.02
RSD (%)	11.11	9.56	11.18	8.41	4.09	3.39	2.33
Total							
Mean RSD (%)	9.04	7.00	7.91	6.98	4.79	4.41	2.92
stdev (RSD)	1.94	2.33	2.94	1.37	0.83	1.10	0.67
Total RSD (%)	6.15						
Greek							
Wavelengths	405	460	525	590	621	660	850
Batch 1							
Reflectance	10.79	19.85	21.80	28.38	35.05	36.60	33.83
stdev	0.43	0.69	1.21	1.36	1.52	1.63	1.54
RSD (%)	4.03	3.48	5.54	4.80	4.34	4.46	4.54
Batch 2							
Reflectance	10.98	19.90	22.18	28.75	35.08	36.38	33.88
stdev	0.25	0.54	0.92	1.38	1.90	2.03	1.55
RSD (%)	2.25	2.73	4.13	4.79	5.42	5.57	4.59
Batch 3							
Reflectance	12.19	21.65	24.23	31.30	39.68	42.46	39.89
stdev	0.99	1.23	2.00	1.81	2.19	2.10	2.20
RSD (%)	8.12	5.67	8.25	5.77	5.51	4.94	5.52
Total							
Mean RSD (%)	4.80	3.96	5.97	5.12	5.09	4.99	4.88
stdev (RSD)	3.01	1.53	2.09	0.57	0.65	0.56	0.55
Total RSD (%)	4.97						

**Table 3:** Performance of three different algorithms used for the prediction of microbial counts/level of freshness in chicken fillets (A. Fresh, B. Thawed and C. The combination of them (All)).

		**PLS-R**		**SVM**		**kNN**	
A. Fresh *	**Samples**	**RMSE**	**R^2^**	**RMSE**	**R^2^**	**RMSE**	**R^2^**
Training	71	0.803	0.889	0.118	0.998	-	-
Validation	10	0.717	0.905	1.128	0.714	-	-
Prediction	25	0.633	0.928	1.015	0.816	0.347	0.979
		**PLS-R**		**SVM**		**kNN**	
**B. Thawed**	**Samples**	**RMSE**	**R^2^**	**RMSE**	**R^2^**	**RMSE**	**R^2^**
Training	61	0.731	0.876	0.135	0.996	-	-
Validation	8	1.049	0.738	1.514	0.282	-	-
Prediction	23	0.787	0.859	1.517	0.467	1.255	0.635
		**PLS-R**		**SVM**		**kNN**	
**C. All**	**Samples**	**RMSE**	**R^2^**	**RMSE**	**R^2^**	**RMSE**	**R^2^**
Training	132	0.850	0.866	0.259	0.987	-	-
Validation	20	0.968	0.773	1.070	0.736	-	-
Prediction	48	0.834	0.863	1.114	0.748	1.350	0.630

* Not frozen/thawed samples.

**Table 4:** Performance of three different algorithms used for the prediction of freshness in chicken fillets (fresh, thawed, combination of them).

Fresh vs. Spoiled (Fresh *)		**Training**	**Validation**	**Prediction**
PLS-DA	Accuracy (%)	98.63	100.00	96.45
	Precision	1.00	1.00	0.933
	Recall	1.00	1.00	0.929
	Specificity	0.976	1.00	1.00
SVM	Accuracy (%)	98.50	100.00	96.43
	Precision	0.974	1.00	1.00
	Recall	1.00	1.00	0.933
	Specificity	0.968	1.00	1.00
kNN	Accuracy (%)	94.10	-	96.43
	Precision	1.00	-	1.00
	Recall	0.900	-	0.933
	Specificity	-	-	1.00
**Fresh vs. Spoiled (Thawed)**		**Training**	**Validation**	**Prediction**
PLS-DA	Accuracy (%)	93.88	100.00	90.00
	Precision	0.944	1.00	0.938
	Recall	0.967	1.00	0.800
	Specificity	0.895	1.00	1.00
SVM	Accuracy (%)	95.90	90.00	90.00
	Precision	1.00	1.00	1.00
	Recall	0.846	0.857	0.875
	Specificity	1.00	1.00	1.00
kNN	Accuracy (%)	83.33	-	95.00
	Precision	0.750	-	1.00
	Recall	0.750	-	0.938
	Specificity	-	-	1.00
**Fresh vs. Spoiled (All)**		**Training**	**Validation**	**Prediction**
PLS-DA	Accuracy (%)	96.64	96.00	96.30
	Precision	0.967	0.909	1.00
	Recall	0.965	0.933	1.00
	Specificity	0.967	1.00	0.926
SVM	Accuracy (%)	96.60	96.00	100.00
	Precision	0.982	0.938	1.00
	Recall	0.947	1.00	1.00
	Specificity	0.984	0.900	1.00
kNN	Accuracy (%)	93.10	-	97.83
	Precision	1.00	-	0.962
	Recall	0.889	-	1.00
	Specificity	-	-	0.952

* Not frozen/thawed samples.

**Table 5:** Performance of three different algorithms used for the discrimination of fresh and frozen/thawed samples testing Greek, Danish and combination of them (All) chicken fillets.

**Fresh vs. Thawed (Greek)**		**Training**	**Validation**	**Prediction**
PLS-DA	Accuracy (%)	89.47	86.67	76.09
	Precision	0.904	0.882	0.700
	Recall	0.930	0.882	0.913
	Specificity	0.837	0.846	0.609
SVM	Accuracy (%)	99.10	92.90	62.50
	Precision	0.986	0.900	0.579
	Recall	1.00	1.00	0.917
	Specificity	0.978	0.800	0.333
kNN	Accuracy (%)	82.10	-	70.83
	Precision	0.882	-	0.647
	Recall	0.833	-	0.917
	Specificity	-	-	0.500
**Fresh vs. Thawed (Danish)**		**Training**	**Validation**	**Prediction**
PLS-DA	Accuracy (%)	81.13	75.00	80.00
	Precision	0.857	0.769	0.800
	Recall	0.826	0.625	0.800
	Specificity	0.800	0.833	0.800
SVM	Accuracy (%)	98.00	96.00	95.00
	Precision	1.00	0.941	1.00
	Recall	0.962	1.00	0.900
	Specificity	1.00	0.889	1.00
kNN	Accuracy (%)	76.00	-	90.00
	Precision	0.706	-	0.864
	Recall	0.923	-	0.950
	Specificity	-	-	0.850
**Fresh vs. Thawed (All)**		**Training**	**Validation**	**Prediction**
PLS-DA	Accuracy (%)	79.76	73.33	63.64
	Precision	0.794	0.750	0.605
	Recall	0.876	0.750	0.788
	Specificity	0.690	0.714	0.485
SVM	Accuracy (%)	85.00	92.50	75.76
	Precision	0.815	0.870	0.718
	Recall	0.957	1.00	0.848
	Specificity	0.895	0.850	0.667
kNN	Accuracy (%)	0.725	-	65.15
	Precision	0.714	-	0.848
	Recall	0.870	-	0.609
	Specificity	-	-	0.750

**Table 6:** Performance of three different algorithms used for the prediction of authenticity (Greek vs. Danish) for fresh (not frozen/thawed), thawed and combination of them (all) chicken fillets.

**Greek vs. Danish (Fresh)**		**Training**	**Validation**	**Prediction**
PLS-DA	Accuracy (%)	100.00	100.00	100.00
	Precision	1.00	1.00	1.00
	Recall	1.00	1.00	1.00
	Specificity	1.00	1.00	1.00
SVM	Accuracy (%)	100.00	100.00	100.00
	Precision	1.00	1.00	1.00
	Recall	1.00	1.00	1.00
	Specificity	1.00	1.00	1.00
kNN	Accuracy (%)	86.40	-	100.00
	Precision	0.875	-	1.00
	Recall	0.933	-	1.00
	Specificity	-	-	1.00
**Greek vs. Danish (Thawed)**		**Training**	**Validation**	**Prediction**
PLS-DA	Accuracy (%)	100.00	100.00	100.00
	Precision	1.00	1.00	1.00
	Recall	1.00	1.00	1.00
	Specificity	1.00	1.00	1.00
SVM	Accuracy (%)	100.00	100.00	100.00
	Precision	1.00	1.00	1.00
	Recall	1.00	1.00	1.00
	Specificity	1.00	1.00	1.00
kNN	Accuracy (%)	88.20	-	100.00
	Precision	0.846	-	1.00
	Recall	1.00	-	1.00
	Specificity	-	-	1.00
**Greek vs. Danish (All)**		**Training**	**Validation**	**Prediction**
PLS-DA	Accuracy (%)	100.00	100.00	100.00
	Precision	1.00	1.00	1.00
	Recall	1.00	1.00	1.00
	Specificity	1.00	1.00	1.00
SVM	Accuracy (%)	100.00	92.50	92.50
	Precision	1.00	0.897	0.897
	Recall	1.00	1.00	1.00
	Specificity	1.00	0.786	0.786
kNN	Accuracy (%)	0.974	-	100.00
	Precision	0.966	-	1.00
	Recall	1.00	-	1.00
	Specificity	-	-	1.00

## Data Availability

The data presented in this study are available upon request from the corresponding author.

## Abbreviations

The following abbreviations are used in this manuscript:

MSI	Multispectral Imaging
PLS-R	Partial Least Squares Regression
kNN	k-Nearest Neighbors
SVM	Support Vector Machine
PLS-DA	Partial Least Squares Discriminant Analysis
PLS	Partial Least Squares
NIR	Near-Infrared
TAC	Total Aerobic Count
RMSE	Root Mean Squared Error
NADH	Nicotinamide Adenine Dinucleotide
